# Multimodal analysis using [^11^C]PiB-PET/MRI for functional evaluation of patients with Alzheimer’s disease

**DOI:** 10.1186/s13550-020-00619-z

**Published:** 2020-03-30

**Authors:** Hidehiko Okazawa, Masamichi Ikawa, Minyoung Jung, Rikiya Maruyama, Tetsuya Tsujikawa, Tetsuya Mori, Mahmudur G. M. Rahman, Akira Makino, Yasushi Kiyono, Hirotaka Kosaka

**Affiliations:** 1 Biomedical Imaging Research Center, University of Fukui, 23-3, Matsuoka-Shimaizuki, Eiheiji-cho, Fukui, 910-1193 Japan; 2 Department of Neurology, Faculty of Medical Sciences, University of Fukui, 23-3, Matsuoka-Shimaizuki, Eiheiji-cho, Fukui, 910-1193 Japan; 3 Department of Psychiatry, Faculty of Medical Sciences, University of Fukui, 23-3, Matsuoka-Shimaizuki, Eiheiji-cho, Fukui, 910-1193 Japan; 4 Department of Biomedical Engineering, Khulna University of Engineering & Technology, Khulna, 9203 Bangladesh

**Keywords:** PET/MRI, Amyloid imaging, Cerebral blood flow, Functional MRI, Quantitative analysis, Multimodal analysis

## Abstract

**Background:**

Multimodal PET/MRI image data simultaneously obtained from patients with early-stage of Alzheimer’s disease (eAD) were assessed in order to observe pathophysiologic and functional changes, as well as alterations of morphology and connectivity in the brain. Fifty-eight patients with mild cognitive impairment and early dementia (29 males, 69 ± 12 years) underwent [^11^C]Pittsburgh compound-B (PiB) PET/MRI with 70-min PET and MRI scans. Sixteen age-matched healthy controls (CTL) (9 males, 68 ± 11 years) were also studied with the same scanning protocol. Cerebral blood flow (CBF) was calculated from the early phase PET images using the image-derived input function method. A standardized uptake value ratio (SUVr) was calculated from 50 to 70 min PET data with a reference region of the cerebellar cortex. MR images such as 3D-T1WI, resting-state functional MRI (RS-fMRI), diffusion tensor image (DTI), and perfusion MRI acquired during the dynamic PET scan were also analyzed to evaluate various brain functions on MRI.

**Results:**

Twenty-seven of the 58 patients were determined as eAD based on the results of PiB-PET and clinical findings, and a total of 43 subjects’ data including CTL were analyzed in this study. PiB SUVr values in all cortical regions of eAD were significantly greater than those of CTL. The PiB accumulation intensity was negatively correlated with cognitive scores. The regional PET-CBF values of eAD were significantly lower in the bilateral parietal lobes and right temporal lobe compared with CTL, but not in MRI perfusion; however, SPM showed regional differences on both PET- and MRI-CBF. SPM analysis of RS-fMRI delineated regional differences between the groups in the anterior cingulate cortex and the left precuneus. VBM analysis showed atrophic changes in the AD group in a part of the bilateral hippocampus; however, analysis of fractional anisotropy calculated from DTI data did not show differences between the two groups.

**Conclusion:**

Multimodal analysis conducted with various image data from PiB-PET/MRI scans showed differences in regional CBF, cortical volume, and neuronal networks in different regions, indicating that pathophysiologic and functional changes in the AD brain can be observed from various aspects of neurophysiologic parameters. Application of multimodal brain images using PET/MRI would be ideal for investigating pathophysiologic changes in patients with dementia and other neurodegenerative diseases.

## Background

Molecular imaging methods such as amyloid/tau PET are very important recently for appropriate diagnosis of patients with dementia, who have cognitive complaints induced by neurodegenerative changes. The relationships among pathophysiologic changes in such diseases have been investigated, and various studies have shown practical aspects of functional imaging methods that have become more important for the assessment of pathologic and physiologic alterations. Conventional morphological imaging methods such as computed tomography (CT) or magnetic resonance imaging (MRI) are also useful for observing atrophic or ischemic changes in the brain, which could be the causes of cognitive impairment. A new hybrid functional brain imaging system combining positron emission tomography (PET) and MRI, the PET/MRI scanner, is very beneficial in this regard, and seems to be ideal in the neuropsychiatric field to apply a molecular and functional imaging approach to the assessment of cerebral pathophysiologic changes [1, 2]. This system has been applied to the studies of neurodegenerative diseases including Alzheimer’s disease (AD) as well as cerebrovascular diseases [3–6]. Several studies showed relationships between changes in metabolism or blood flow and functional or morphological MRI information; however, reports of integrated analysis using amyloid/tau imaging and functional MRI are very few.

The Alzheimer’s Disease Neuroimaging Initiative (ADNI) project (http://adni.loni.usc.edu/) and similar local multicenter projects have accumulated large amount of patient data that are freely accessible from everywhere in the world. Multimodality image data sets such as PET and MRI are also available, allowing analysis of the brain pathophysiology of AD patients over a wide range of neurodegenerative stages [7]. However, the multimodal brain images in these projects were acquired using various scanning protocols and image qualities for both PET and MRI, because the image reconstruction methods were not unified. Thus, the image quality depended on the methods of individual hospitals and institutes, which may affect quantitative evaluation and statistical analyses. In the present study, [methyl-^11^C]-2-(4′-methylaminophenyl)-6-hydroxybenzothiazole ([^11^C]PiB) was used for assessment of amyloid deposition in the cerebral cortex, to distinguish AD patients from other dementias, and quantitative PET data were evaluated by absolute values. A new method for quantitative cerebral blood flow (CBF) calculation was proposed here using the initial frames of dynamic [^11^C]PiB PET/MRI data, and regional CBF distribution were compared with MRI perfusion images. Functional aspects of the brain were directly compared between PET and MRI, including perfusion, standard uptake value (SUV) for [^11^C]PiB-PET, neuro-functional connectivity using resting-state functional MRI (RS-fMRI), and morphological tissue volume with three-dimensional (3D) MRI. A single data set was obtained from each patient simultaneously during dynamic PET/MRI acquisition, which provides a high-quality evidence of pathophysiologic changes. The purpose of this study was to evaluate various aspects of the neuro-functional changes in dementia with multiple parameters reflecting neuro-physiologic activity using the practical multimodal imaging method of PET/MRI.

## Material and methods

### Subjects

Fifty-eight patients with mild cognitive impairment or early dementia (29 males and 29 females, 69 ± 12 years) were studied using [^11^C]PiB PET/MRI to evaluate amyloid deposition in the brain. We made a differential diagnosis of dementia for all patients and determined the indication of medications. They approved our investigation for assessment of pathophysiologic changes in dementia and neuro-degenerative changes. Sixteen age-matched healthy volunteers (9 males and 7 females, 68 ± 11 years) also underwent [^11^C]PiB-PET/MRI as a control group (CTL). The study was approved by the Ethics Committee of the University of Fukui, Faculty of Medical Sciences, based on its guidelines (Ethical Guidelines for Medical Science Research with Humans) as well as the Helsinki Declaration of 1975 (revised in 1983). Written informed consent was obtained from each participant involved in this study. All patients were able to give their consent on their own.

### PET/MRI scanner

A whole-body PET/MRI scanner (Signa PET/MR, ver. 26, GE Healthcare, Milwaukee, WI, USA) was used for simultaneous PET and MRI data acquisition [8]. The scanner permits PET acquisition of 89 image slices in 3D mode, with a slice thickness of 2.45 mm. Performance tests showed the intrinsic resolution of PET images to be 4.2–4.3 mm full width at half maximum (FWHM) in the transaxial direction. The PET/MRI scanner was calibrated with a dose-calibrator (CRC-12, Capintec Inc., NJ, USA) beforehand using a pool phantom and ^18^F-solution, according to the scanner manufacturer’s guidelines [9].

### PET and anatomical MRI acquisition

The patients underwent brain PET/MRI scans using a standard head coil (8-channnel HD Brain, GE Healthcare) for simultaneous PET and MRI acquisition. A 70-min list-mode 3D PET scan in time-of-flight (TOF) acquisition mode was started at the time of a bolus tracer injection of 700–750 MBq [^11^C]PiB via the antecubital vein. During the PET scan, a 3D radial MR acquisition for the zero-echo time (ZTE) method in the axial direction was performed for attenuation correction (AC) of PET data with the following parameters: FOV 264 mm, matrix 110 × 110 × 116, voxel size 2.4 × 2.4 × 2.4 mm^3^, flip angle 0.8°, number of excitations 4, bandwidth ± 62.5 kHz, and acquisition time of 41 s [10, 11]. In the ZTE-AC method, the following process was used to create the MR-AC map based on the previous study [10]. Briefly, the first step was pre-filtering and histogram-based normalization, followed by an intensity-based segmentation of the head, bias correction, identification of voxels affected by partial volume effects, and segmentation of the sinus, bone, and cavity masks. A pseudo-CT map was then generated with bone tissue linearly scaled based on the ZTE intensity. This mapping was determined by fitting of registered CT and ZTE data in the bone density range. To convert the pseudo-CT into a MRAC map, the images were re-sampled with a 60 × 60 × 25 cm^3^ field of view (FOV) in 128 × 128 × 89 matrix, and finally re-scaled to 511 keV attenuation coefficients.

3D TOF MR angiography (MRA) and other anatomical MR images such as T2-weighted (T2WI) and FLAIR, etc. were acquired in the same position during the PET scan. High resolution 3D-T1-weighted (T1WI) anatomical MRI was also collected using the following parameters: repetition time (TR) = 6.38 ms; echo time (TE) = 1.99 ms; flip angle = 11°; FOV = 256 mm; 256 × 256 matrix; 172 slices; voxel dimension = 1.0 × 1.0 × 1.0 mm^3^.

### Resting-state fMRI and diffusion tensor image acquisition

Functional MR images for RS-fMRI analysis were acquired with a T2-weighted gradient-echo echo-planar imaging (EPI) sequence during the dynamic [^11^C]PiB PET data acquisition. A total of 201 volumes were collected in an image acquisition time of 7 min 42 s. Each volume consisted of 40 slices, with a thickness of 3.5 mm and a 0.5-mm gap to cover the entire brain using the following parameters: TR = 2300 ms; TE = 30 ms; flip angle = 81°; FOV = 192 × 192 mm; 64 × 64 matrix; pixel dimension = 3 × 3 mm^2^. The subjects were instructed to stay awake but close their eyes and think of nothing in particular. Head movement was minimized by the placement of a specific pillow and sponge around their head.

MR diffusion tensor image (DTI) acquisition for fractional anisotropy (FA) analysis was followed by RS-fMRI during the PET scan. Diffusion-weighted images (DWI) were acquired using single-shot EPI (TE = minimum; TR = 9327 ms; FOV = 240 mm; 240 × 240 matrix; pixel size = 1.9 × 1.9 mm^2^; 45 axial slices; slice thickness/gap = 3.0 mm/0 mm) with 30 distributed isotropic orientations for the diffusion-sensitizing gradients at b-values of 1000 s/mm^2^ and 0.

### PET reconstruction and image calculation of CBF and SUV

Dynamic PET images were reconstructed from the PET data using the 3D ordered-subset expectation maximization (OSEM) method and point spread function (PSF) modeling algorithm in 39 frames of 12 × 5 s, 6 × 10 s, 3 × 20 s, 4 × 30 s, 5 × 60 s, 4 × 5 m, and 4 × 10 m. Time-activity curves were obtained from the dynamic PET data using the image-derived input function (IDIF) method described previously [9, 12]. The initial 21 frames for 3 min after tracer injection were used for calculation of CBF. The IDIF input function and dynamic PET data reconstructed with the OSEM parameter set of subset, 28; iteration, 3; transaxial post-Gaussian filter cutoff, 3 mm in 256 mm FOV and 2 × 2 mm^2^ pixel size were applied. The decay of radioactivity in the dynamic PET data was corrected to the starting point of each scan.

Details of the IDIF estimation from PET/MRI data are described elsewhere [9]. In brief, the average image of the initial phase (10–40 s) of dynamic PET data was used to extract voxels only inside the ICA, where the 30 most intensely radioactive voxels were selected as the volume of interest (VOI) mask at the region of the cavernous part of the ICA. Individual 3D TOF-MRA images were used to confirm the location of the arterial VOI mask precisely on the ICA. Time-activity curves for IDIF were obtained by applying the ICA VOI masks on the dynamic PET data [9, 12]. Arterial input functions for the initial 3 min were estimated from IDIF total blood radioactivity. Since the radioactivity fraction of unmetabolized tracer is assumed to be 0.95 during the initial time frames of dynamic data [13], each IDIF curve was corrected for the plasma input function (C_p_) using this fraction.

CBF images (mL/min/100 g) were calculated based on a one-tissue compartment model using the three-weighted integral calculation method [14, 15]. In this method, an image of *K*_1_, the tissue influx rate constant of PET tracer, was calculated first, and the image was then converted to a CBF image using an extraction fraction (E) of 0.65 which was estimated from previous reports where *K*_1_ of [^11^C]PiB was calculated [16, 17], and CBF values in healthy controls on PET/MRI in our previous study [9], using an equation of *K*_1_ = *E* × CBF. The dispersion in the peripheral arteries was automatically corrected in the program with a fixed constant of 4 s. No delay correction of tracer arrival time between C_p_ and the major cerebral arteries was needed because the input function was obtained from the same dynamic PET data at the skull base [9].

SUV images were also calculated from static images of the 50–70 min time frame of the list-mode PET data. Each static image was corrected for the individual patient’s body weight (BW) and injection dose (ID) as follows: SUV image = PET image/(ID/BW). To compare [^11^C]PiB accumulation in the brain, each SUV image was normalized by the individual cerebellar cortical SUV value and saved as the SUV ratio (SUVr) image [17].

### Arterial spin labeling (ASL) method for CBF

A pseudo-continuous ASL (pCASL, GE’s product version) prepped 3D spiral fast spin-echo (FSE) acquisition combined with background suppression was used for calculation of MRI perfusion images [18]. Details of the method are described elsewhere [19]. In brief, the labeling duration for ASL preparation was 1.5 s, and the 3D-FSE readout signal was obtained with an interleaved stack of 7-arm acquisitions for each excitation at each of 36–42 centrically ordered slice encodes. The labeling slab is automatically set at a level 2 cm inferior to the scan range, and images were acquired with the following parameters: 240 mm FOV; 128 × 128 matrix; in-plane resolution 1.8 mm; slice thickness 4.5 mm; fixed post-labeling delay (PLD) 2.0 s [19]. The number of excitations for the acquisition was three, and the total scan duration was 4 min 10 s. For blood flow quantification, an approximate proton density weighted image was also obtained with the same acquisition parameters. CBF (mL/min/100 g) calculation was performed using the previously defined model [20, 21].

### RS-fMRI data preprocessing

The RS-fMRI data were analyzed using Statistical Parametric Mapping (SPM12; http:// www.fil.ion.ucl.ac.uk/spm/, Wellcome Trust Centre for Neuroimaging, London, UK) via Matlab 2016 (Mathworks, Natick, MA, USA), a data processing assistant, and RS-fMRI software DPARSF [22]. First, the initial 10 volumes were discarded, and slice-timing correction was performed, followed by spatial realignment of 191 volumes to the mean volume. The signal from each slice was realigned temporally to that obtained from the middle slice using sinc interpolation. The resliced volumes were normalized to the Montreal Neurological Institute (MNI) space with a voxel size of 2 × 2 × 2 mm^3^ using individual 3D-T1WI images in the same location and the template provided by SPM12. The normalized images were spatially smoothed with a 6-mm Gaussian kernel [23, 24]. Next, the linear trend in the time series was removed, the non-neural noise in the time series was controlled, and several sources of spurious variance (e.g., the Friston 24-parameter model, white matter signals, and cerebrospinal fluid signals) were removed from the data through linear regression [25, 26]. To control for motion confounds in the RS-fMRI data, we investigated the effects of head motion by computing the mean frame-to-frame root mean squared motion, and frame-wise displacement obtained during the realignment process [27, 28]. The parametric images of amplitude of low-frequency fluctuation (ALFF) and functional ALFF (fALFF) were calculated. In the present study, fALFF, the most common parameter in RS-fMRI analysis, was used because it enhances the amplitude by reducing noise signals of ALFF.

### 3D-T1WI volume-based analysis

Voxel-based morphometry (VBM) analysis using 3D-T1WI data was performed using the standard Diffeomorphic Anatomical Registration Through Exponentiated Lie Algebra (DARTEL) processing pipeline in SPM12 [29]. All the scans were checked for artifacts and poor image quality before data processing. Subsequently, each image was reoriented in order to set the anterior commissure at the origin of the MNI coordinate space. The 3D-T1WI images were then parcellated into different tissue classes—gray matter (GM), white matter (WM), and non-brain voxels such as cerebrospinal fluid (CSF)—and skull, based on separate tissue probability maps for each tissue class using the segmentation approach implemented in SPM12. The DARTEL algorithm was used to generate a study-specific template, and the resulting flow fields generated by DARTEL were used to obtain GM images of each subject; these images were spatially normalized in the MNI space, modulated, resliced (1.5 mm isotropic voxels), and smoothed with an 8-mm FWHM.

The volume of the hippocampus region was separately analyzed using a free software program, the voxel-based specific regional analysis system for Alzheimer’s disease (VSRAD) based on SPM and DARTEL [30, 31]. The GM, WM, or CSF images segmented from 3D-T1WI images of all subjects were anatomically standardized to a customized template of GM, and then smoothed using an 8-mm FWHM isotropic Gaussian kernel. VSRAD provides regional *Z*-scores, defined as: ([control mean]−[individual value])/(control SD), for GM atrophy in each subject compared with that of the normal database of GM.

### FA image analysis

We used Freesurfer 6.01 to reconstruct each participant’s T1WI image to obtain GM and WM volumes by discriminating the cortical and subcortical regions in individual subjects [32]. The DTI data were submitted to analysis using TRActs Constrained by UnderLying Anatomy (TRACULA) [33], an automated global probabilistic tractography tool in Freesurfer, which delineates 18 WM pathways in DWI data. The method relies on prior knowledge of pathway anatomy to reconstruct the tracts based on a manually labeled training set of subjects. Four diffusion measures (FA, MD, RD, and AD) were extracted at each voxel, weighted by the pathway probability in each of the 18 WM pathways by thresholding the pathway distribution at 20% of its maximum value for each subject. The FA data were used for analysis in this study.

### Statistical analyses

We used PMOD software (version 3.9; PMOD Technologies Ltd., Zurich, Switzerland) to compare the regional CBF and SUVr values between AD and CTL. Image parcellation in the PMOD software performed segmentation of brain regions based on the individual 3D-T1WI MRI image data. SUVr and CBF values from [^11^C]PiB-PET and MRI-ASL were calculated according to the regions of interest (ROIs) determined on the 3D-T1WI. The location of PET and MRI was exactly the same because of simultaneous acquisition on the PET/MRI system. Each regional value was the average of ROIs in the same region. Repeated measures analysis of variance (ANOVA) with a post hoc paired *t* test was applied to analyze differences in the values of regional CBF, SUVr, and other clinical data between AD and CTL. *P* < 0.05 was considered to be significant.

A general linear model in SPM12 was used to evaluate differences between the two groups of AD and CTL for CBF from PET and ASL-MRI, GM volume from VBM analysis, and fALFF from RS-fMRI. In VBM analysis, GM volume and age were included as nuisance covariates. In fALFF analysis of the RS-fMRI data, each time series at each voxel as a fraction was calculated between the sum of amplitudes of the band ranging from 0.01 to 0.08 Hz, and subject-level voxel-wise fALFF maps were standardized into subject-level *Z*-score maps using the DPARSF toolbox. For group comparisons, each image pertaining to *z* values was entered into SPM12 with two-sample *t* tests. We applied a statistical height threshold of *P* < 0.005 uncorrected and a cluster size of 50 voxels and greater, and the cluster-level of *P* < 0.05 was then applied for assessment of significant differences. For assessment of WM function, the brain FA data of all subjects were realigned into the FMRIB58_FA standard space template using the “dtifit” function in FSL (FMRIB Software Library; https://www. fmrib.ox.ac.uk/fsl/). We used voxel-wise analysis with tract-based spatial statistics (TBSS) to generate whole-brain statistical maps of FA to observe differences between AD and CTL.

## Results

Twenty-seven of the 58 patients studied were diagnosed as early stage Alzheimer’s disease defined by positive cortical [^11^C]PiB accumulation and clinical findings, and thus, a total of 43 subjects were analyzed and compared in this study. Clinical information of the two groups are given in Table 1. There were no differences in age range, mean age, and gender ratio for the two groups. The AD group showed a significant decrease in MMSE score (*P* < 0.0001). Although the hippocampus volumes in AD patients tended to decrease in the *Z*-score of VSRAD, the difference was not significant in comparison with CTL (*P* > 0.05). Twenty-one of the 27 patients underwent perfusion SPECT prior to [^11^C]PiB-PET/MRI, and 14 of them were suggested to be early AD based on the statistical mapping analysis.

**Table 1:** Clinical information

Group	Age (years old)	Sex	VSRAD	Perfusion SPECT	MMSE	ADAS
AD (*N* = 27)	69 ± 12	M 13 F 14	1.86 ± 0.99	s/o DAT 14 s/o FTD 4 WNL 3	24 ± 3*	15 ± 8
CTL (*N* = 16)	68 ± 11	M 9 F 7	1.27 ± 0.78	–	29 ± 1	–

Perfusion SPECT was performed in 21 patients using *N*-isopropyl-p-[^123^I]iodo-amphetamine (IMP)

*AD* Alzheimer’s patients, *CTL* healthy controls, *VSRAD* voxel-based specific regional analysis system for Alzheimer’s disease, *MMSE* Mini-Mental State Examination, *ADAS* Alzheimer’s Disease Assessment Scale-Cognitive Subscale, *DAT* dementia of Alzheimer’s type, *FTD* frontotemporal dementia

*P < 0.00001

**Fig. 1:**
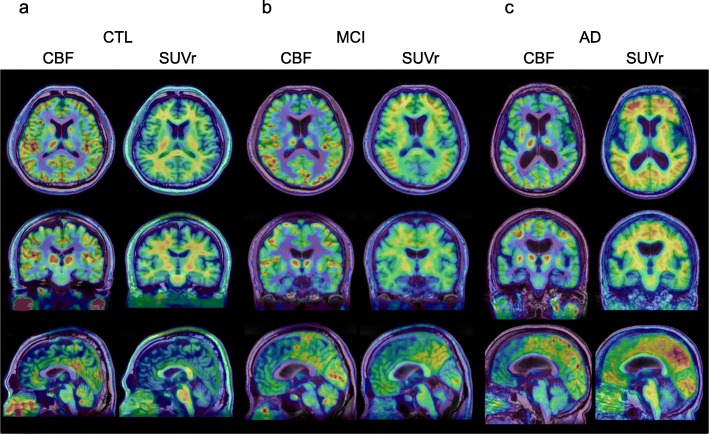
Representative CBF (left) and SUVr (right) images of healthy control (CTL) (**a**), MCI (**b**), and early AD (**c**) calculated from [^11^C]PiB-PET. MMSE scores were 30, 29, 25, respectively. SUVr images showed a gradual increase of cortical [^11^C]PiB accumulation according to severity of the disease, but CBF values were close between controls and MCI patients. CBF (mL/min/100 g) images showed similar distribution between MCI and CTL, while early AD patients showed slight decrease in frontal and temporal lobes. Note the posterior cingulate cortex and the precuneus did not show CBF decrease and gradual SUVr increase in the AD patient

**Fig. 2:**
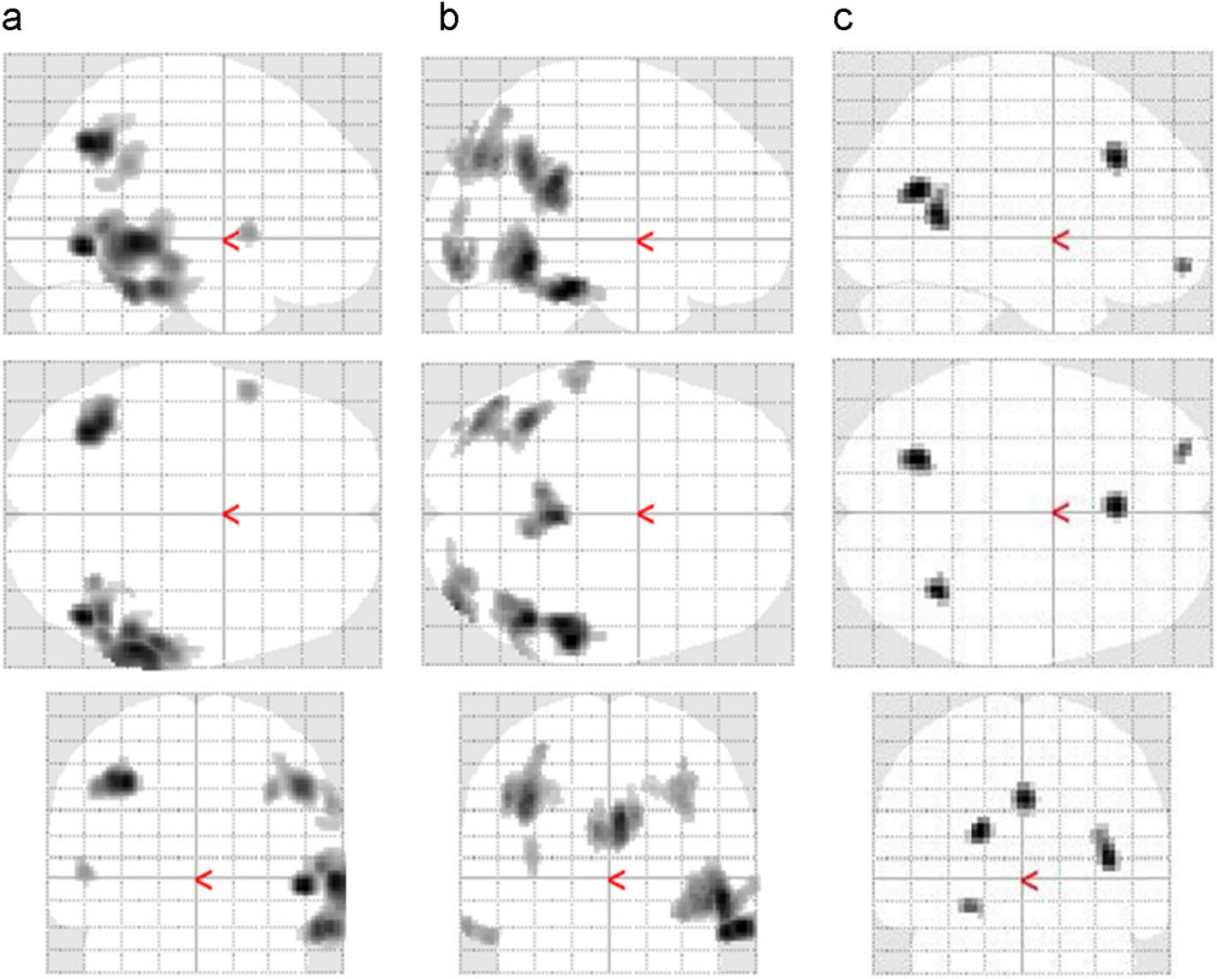
SPM comparison of AD and CTL groups for PET- (**a**) and ASL-CBF (**b**), and RS-fMRI (**c**). All subject data of 27 AD and 16 CTL were applied in PET-CBF analysis, but only 25 AD datasets could be used for ASL-CBF comparison because of poor image quality due to artifacts. SPM analysis showed similar results of CBF reduction in AD group for both PET- and ASL-CBF comparison. Note the difference in the posterior cingulate cortex was observed only in the ASL-CBF analysis. RS-fMRI analysis showed significant decreases in the AD group in the anterior cingulate cortex and in the inferior part of the left precuneus (*P* < 0.005, cluster-level *P*_uncorr_ < 0.05). No significant increases were observed in AD for all parameters

Figure 1 shows representative cases of healthy control, MCI (prodromal AD), and early AD patients. The [^11^C]PiB accumulation intensity gradually increased according to severity of the disease, but CBF values were close between controls and MCI patients. Table 2 shows regional PET-CBF and SUVr values for the CTL and AD groups. The average regional PET-CBF values of the CTL group were similar to our previous [^15^O]water PET studies [9]. The regional PET-CBF values of the AD group tended to be smaller in all regions than those of the CTL group, although only the bilateral parietal and right temporal lobes showed significantly lower CBF values compared with the CTL group (*P* < 0.05). SPM comparison of PET-CBF also showed corresponding brain regions to be significantly different in the cluster-level (*P*_uncorr_ < 0.05, Fig. 2a).

**Table 2:** Regional PET-CBF (mL/min/100 g) and SUVr values (mean ± SD)

	CTL right	CTL left	AD right	AD left
PET-CBF				
Frontal	41.6 ± 4.5	39.9 ± 4.0	38.7 ± 8.7	37.7 ± 8.0
Temporal	39.9 ± 3.0	36.6 ± 3.1	34.5 ± 8.6*	32.2 ± 7.4
Parietal	44.3 ± 5.8	41.7 ± 4.7	37.8 ± 10.6*	36.3 ± 9.3*
Occipital	44.0 ± 5.8	42.3 ± 4.7	39.7 ± 10.4	38.4 ± 9.4
Cerebellum	45.4 ± 5.9	43.9 ± 5.9	42.7 ± 9.4	41.6 ± 8.7
PiB-SUVr				
Frontal	1.02 ± 0.18	1.04 ± 0.19	1.74 ± 0.28^†^	1.77 ± 0.32^†^
Temporal	1.00 ± 0.12	0.99 ± 0.12	1.54 ± 0.28^†^	1.54 ± 0.27^†^
Parietal	1.01 ± 0.16	1.03 ± 0.19	1.79 ± 0.30^†^	1.81 ± 0.31^†^
Occipital	0.99 ± 0.12	1.02 ± 0.12	1.50 ± 0.24^†^	1.51 ± 0.21^†^

*CTL* healthy control, *AD* Alzheimer’s disease, *PET-CBF* cerebral blood flow calculated from early phase PiB-PET data, *SUVr* standardized uptake value ratio

*P < 0.05

^†^*P* < 0.00001 compared with the corresponding region of the CTL group

Twenty-five CBF images of ASL-MRI (MRI-CBF) were analyzed in this study because the image quality of the other two patients was not good enough for comparison due to artifacts from the metal teeth or very low ASL signals in the posterior circulation. The latter sometimes occurs because of elongation of the vertebral arteries. The whole brain means of MRI-CBF for the CTL and AD groups were 48.8 ± 10.1 and 45.4 ± 10.3 (mL/min/100 g), respectively. The values tended to be greater in MRI-CBF than in PET-CBF, although they were not significantly different because of greater variances of MRI-CBF compared with PET-CBF. The greater variances also prevented demonstration of regional differences in quantitative MRI-CBF values between the groups. However, SPM analysis after global normalization of MRI-CBF showed similar results to PET-CBF analysis in the comparison of 25 AD patients and 16 healthy controls (Fig. 2b).

**Fig. 3:**
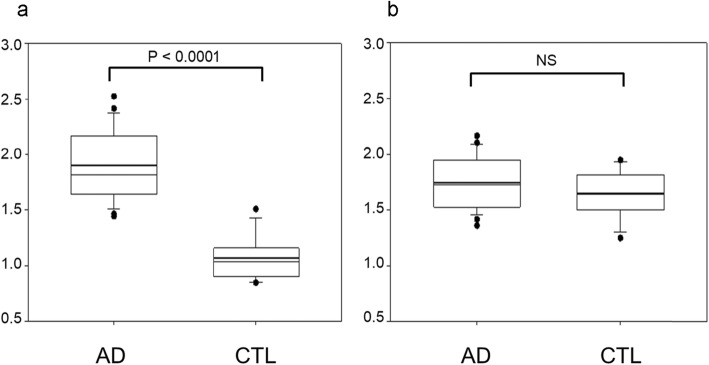
Average SUVr values of [^11^C]PiB in cerebral cortex (**a**) and white matter (**b**). The AD group showed significantly greater cortical SUVr than the CTL group (*P* < 0.0001); however, white matter accumulation showed no difference between the AD and CTL groups (NS: *P* > 0.05). Note mean values of CTL were lower in the cerebral cortex than in the white matter

**Fig. 4:**
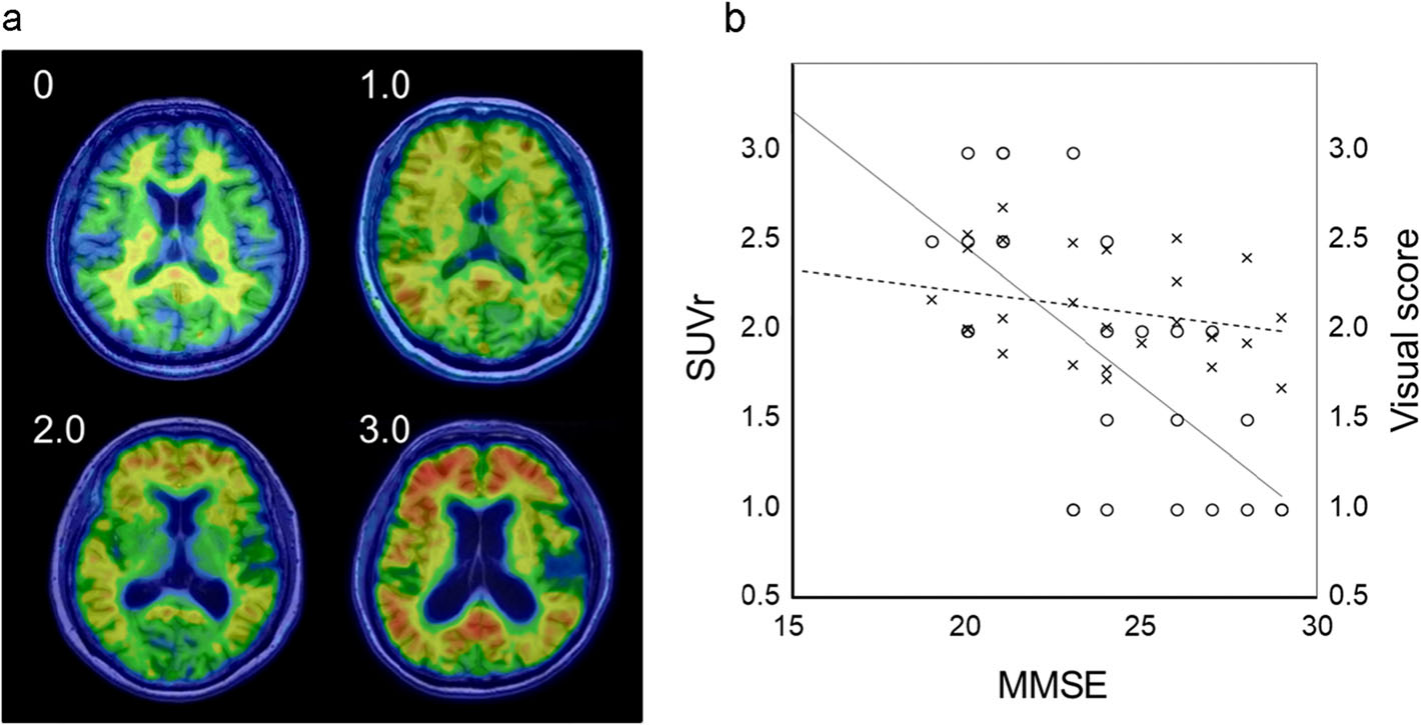
Visual scores for assessment of cortical [^11^C]PiB deposition (**a**) defined as 0, negative; 1, slightly positive; 2, moderate; and 3: severe accumulation. The graph shows correlation between MMSE scores and [^11^C]PiB accumulation intensity determined by SUVr (crossing) and visual scores (open circle) (**b**). The plots of MMSE vs. SUVr did not show significant correlation (dotted line, *r* = 0.30, *P* > 0.05), while those of MMSE vs. visual score showed a significant correlation (solid line, *r* = 0.71, *P* < 0.0005)

[^11^C]PiB SUVr values in all brain regions of the AD group were significantly greater than those of the CTL group (Table 2), and the average cortical SUVr values were significantly different between the groups (*P* < 0.0001), especially in the posterior cingulate cortex (PCC) (AD vs. CTL: 2.1 ± 0.4 vs. 1.1 ± 0.2 for both sides, *P* < 0.00001); however, WM accumulation showed no difference between the groups (Fig. 3). Figure 4 shows the correlation between cognitive deterioration evaluated by MMSE score and the cortical [^11^C]PiB accumulation intensity. Visual score was defined based on the visual impression of two nuclear medicine physicians for intensity and the size of tracer deposition in 4 scores: 0, negative cortical uptake; 1, slight cortical accumulation close to white matter uptake; 2, moderate cortical accumulation; and 3, severe cortical accumulation (Fig. 4a). Scatter plots for MMSE vs. cortical SUVr means and for MMSE vs. visual scores are given in Fig. 4b. The cortical SUVr mean did not show a significant correlation (*r* = 0.30, *P* > 0.05), but the visual score did (*r* = 0.71, *P* < 0.0005).

**Fig. 5:**
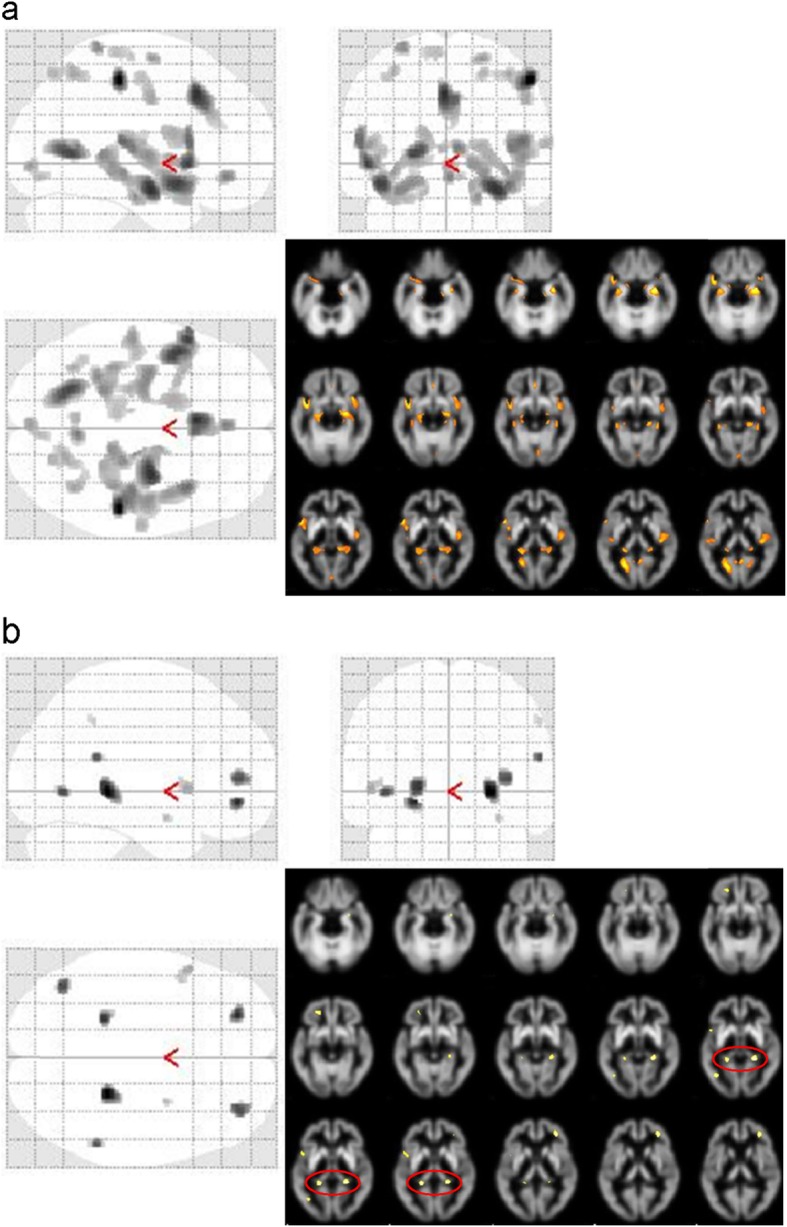
VBM analysis for assessment of atrophic changes in AD patients. Comparison with preserved concentration showed many regional decreases in the AD group (**a**); however, if the differences of the whole brain size were corrected (preserved amount), only a few regions showed differences including the posterior part of the bilateral hippocampus (red circle, peak-level *P* < 0.001, cluster-level *P*_uncorr_ < 0.05) (**b**)

Resting state brain functions were evaluated using fALFF data obtained from RS-fMRI EPI images. SPM analysis showed differences between the groups, where the anterior cingulate cortex (ACC) and the inferior part of the left precuneus had a decrease in the AD group (Fig. 1c, peak-level *P* < 0.005, cluster-level *P*_uncorr_ < 0.05). Other regions did not show any differences between the groups. In analysis of FA data, there were no differences between the two groups. VBM analysis showed atrophic changes in the several brain regions of the AD patients (Fig. 5). The comparison with preserved concentration showed differences in various regions (Fig. 5a); however, if the differences of individual whole brain size were corrected (preserved amount), only a few regions showed differences, including the posterior part of the bilateral hippocampus (Fig. 5b, peak-level *P* < 0.001, cluster-level *P*_uncorr_ < 0.05).

## Discussion

The present study evaluated various parameters related to pathophysiological and functional changes in the brains of AD patients obtained by PET/MRI. AD patients were defined by the results of [^11^C]PiB-PET images and clinical information, and the AD group was compared with the CTL group which consisted of age-matched healthy volunteers. The AD group showed CBF decrease in the bilateral parietal and right temporal lobes, indicating the typical decrease patterns observed in AD patients [34–36]. Both PET- and MRI-CBF delineated these differences; however, some patients had a low-quality ASL images due to teeth metal artifacts or arterial elongation, which could not be used for analysis. VBM analysis delineated a decrease in the bilateral hippocampus in AD patients, which is also known as a common finding. However, after the whole brain size correction, only a small part of the hippocampus showed a significant difference (Fig. 5b). AD patients showed these changes in the brain even in the early stage of disease. In contrast, SUVr values of the WM and FA analysis did not differ between the groups. Since WM accumulation of [^11^C]PiB is considered to reflect myelination of the brain [37–39], the WM function seemed to be preserved in patients of the present AD group.

**Fig. 6:**
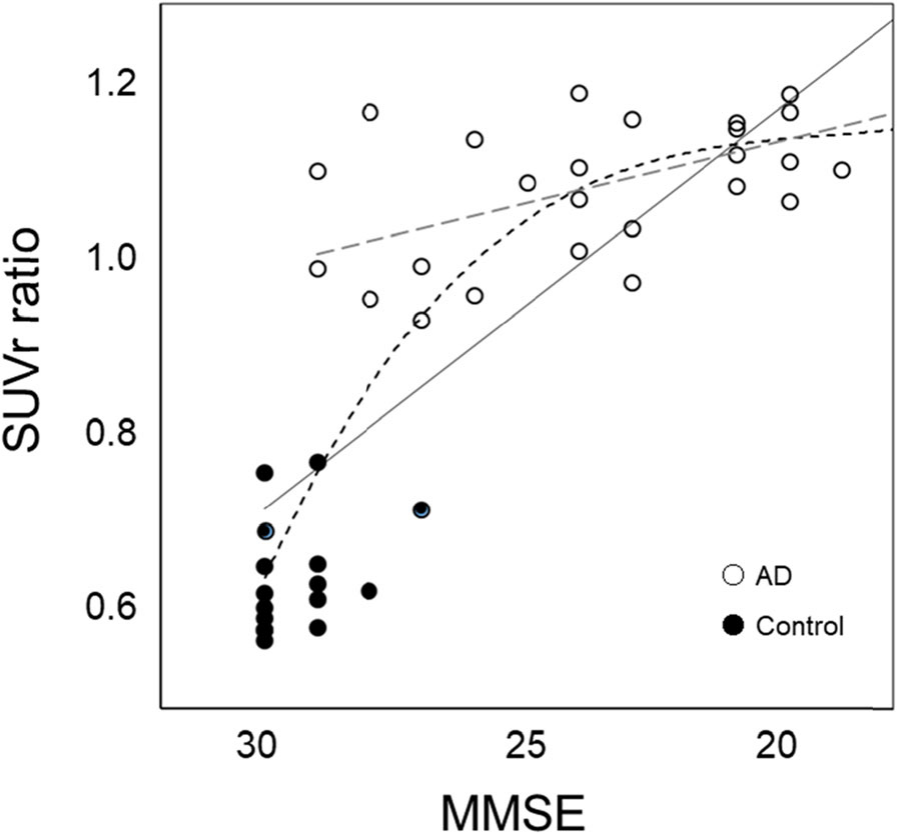
Scatter plot of SUVr ratio calculated by GM-SUVr/WM-SUVr and MMSE score. AD patients (open circle) showed a significant correlation (gray dotted line, *r* = 0.59, *P* < 0.01) and significantly greater ratio than CTL. All plots for both groups also showed a significant correlation (solid line, *r* = 0.81, *P* < 0.0001). Black dotted line represents theoretical assumption line from previous reports

Cortical SUVr values of [^11^C]PiB showed a tendency of negative correlation with cognitive impairment measured by MMSE, while visual scores were well correlated with MMSE (Fig. 4b). The latter result reflects our impression that [^11^C]PiB accumulation seemed to be related with the severity of dementia. When SUVr values were corrected by individual WM SUVr values (cortical SUVr ratio) and plotted with all AD and CTL data as in Fig. 6, a sigmoidal change in [^11^C]PiB accumulation was seen as a function of cognitive decline. This curve is similar to the well-known changes in biomarkers according to AD stage progression [40]. The cortical SUVr ratio showed a significant correlation with MMSE score similar to the visual score graph (Fig. 4b), reflecting contrast of gray and white matter, and clearly discriminated AD patients from CTL. To confirm the time course of AD progression, many CTL subjects should be followed up for a long time as a cohort study.

The most important feature of PET/MRI scans would be obtaining simultaneous multimodal images for both morphological and functional information. The brain images would be one of the most suitable targets for PET/MRI systems. PET molecular imaging provides pathologic information of abnormal aggregation of proteins such as amyloid and tau as well as physiological information of CBF obtained from the initial phase of dynamic PET scans. MRI scans also provide functional images other than anatomical and histopathologic information such as perfusion, WM function, and default-mode network. The combination of these multimodal parameters would provide ideal information to evaluate brain pathophysiology in patients with neurodegenerative diseases.

A dynamic [^11^C]PiB PET scan provides not only quantitative values reflecting cortical amyloid deposition, but also CBF values calculated from initial PET frame data. Permeability of the tracer is considered to be high enough to calculate CBF [16, 41], although there is no definite information about the extraction fraction of the tracer in the human brain. Previous report showed results from simulation analysis where the influx rate-constant of *K*_1_ was about 0.27–0.32 for healthy controls [17], and extraction fraction of 0.77 in the monkey brain [16]. However, CBF values in monkeys were very low (30–38 mL/min/100 g) compared with human brain probably due to effects of anesthesia. Since *K*_1_ = *E* × *F*, where *E* is extraction of the tracer and *F* is blood flow, we assumed that the extraction should be about 0.65, based on the average CBF values in Japanese healthy volunteers [6, 9, 42]. Because the metabolite ratio of [^11^C]PiB is considered to be about 0.95 during the initial scanning time after the injection [13], we corrected the initial arterial input function obtained from the IDIF method using this fixed ratio. The PET-CBF values were very close to our previous studies in CTL after the correction described above, although the present study tended to show slightly lower values because of the higher age range compared with our previous studies [6, 9].

MRI-CBF tended to show greater regional values compared with PET-CBF, which was similar to the results of our previous [^15^O]water PET/MRI study [9]. SPM analysis showed almost the same findings of group differences in CBF obtained from [^11^C]PiB-PET and ASL-MRI after global normalization. However, only MRI-CBF comparison showed a significant difference in the PCC, one of the common regions showing declining glucose metabolism and blood flow in AD patients [34–36, 43]. [^11^C]PiB accumulation in the PCC was significantly greater in SUVr images of the AD group compared with CTL (Fig. 1), suggesting that amyloid binding of the tracer may have been affected even in the early phase kinetics of PET data. Since PET-CBF is calculated based on one-tissue but not two-tissue compartment analysis, tracer binding to Aβ aggregation may influence *K*_1_ values. According to this assumption, the MRI perfusion images may have accurately reflected regional CBF in this study. However, it should be noticed that elderly people sometimes have poor image quality on ASL-MRI because of arterial elongation caused by sclerotic changes, especially in the posterior circulation from the vertebral arteries, as observed in a few patients in this study. Furthermore, variances in MRI-CBF were greater than those in PET-CBF because several patients showed very low MRI-CBF values due to an arterial arrival time longer than 2 s which was used as PLD in this study. The fixed PLD for ASL acquisition may not have been appropriate for these elderly people, and an advanced version of ASL with delay correction for the arterial arrival time is expected [9].

RS-fMRI showed the group differences in the ACC and inferior part of precuneus. The changes in these areas were different from the results of CBF and VBM because the physiological implications of each parameter are different. Since fALFF reflects functional co-fluctuation in the brain, fALFF of the AD brains may have declined compared with CTL in those regions. The ACC is related to positive motivation, and AD or other dementia patients tend to show apathy as one of their clinical symptoms [44, 45]. We were able to observe well-known regional differences in CBF and VBM analysis, but these images did not delineate differences in other functional activities as presented in RS-fMRI analysis. Multimodal assessment of the same disease allows us to observe various neuro-physiological parameters, which may elucidate different pathophysiologic changes in the disease from multiple aspects.

## Conclusion

[^11^C]PiB-PET/MRI scans provided multiple parameters obtained from a single scan, reflecting various neurophysiologic aspects such as CBF, cortical volume, intra-cerebral neuronal networks, and WM functions, in addition to the pathologic alteration of amyloid deposition. Quantitative assessment of functional and pathophysiologic changes is also available through various recently developed methods for PET and MRI. Multimodal brain images using PET/MRI are ideal for investigating various pathophysiologic changes in patients with dementia and other neurodegenerative diseases.

## Data Availability

The datasets used and/or analyzed during the current study are available from the corresponding author on reasonable request.

## Abbreviations

3D: Three-dimensional
AC: Attenuation correction
ACC: Posterior cingulate cortex
AD: Alzheimer’s disease
ADNI: Alzheimer’s Disease Neuroimaging Initiative
ALFF: Amplitude of low-frequency fluctuation
fALFF: Functional ALFF
ASL: Arterial spin labeling
BW: Body weight
CTL: Healthy controls
CBF: Cerebral blood flow
CSF: Cerebrospinal fluid
CT: Computed tomography
DARTEL: Diffeomorphic Anatomical Registration Through Exponentiated Lie Algebra
DTI: Diffusion tensor image
DWI: Diffusion-weighted images
EPI: Echo-planar imaging
FA: Fractional anisotropy
FOV: Field of view
FSE: Fast spin echo
FWHM: Full width at half maximum
GM: Gray matter
ID: Injection dose
IDIF: Image-derived input function
MRI: Magnetic resonance imaging
OSEM: Ordered subset expectation maximization
pCASL: Pseudo-continuous ASL
PCC: Posterior cingulate cortex
PET: Positron emission tomography
[^11^C]PiB: Pittsburgh compound-B = [methyl-^11^C]-2-(4′-methylaminophenyl)-6-hydroxybenzothiazole
PLD: Post-labeling delay
PSF: Point spread function
RS-fMRI: Resting-state functional MRI
SPM: Statistical Parametric Mapping
SUV: Standard uptake value
SUVr: SUV ratio;
T1WI: T1-weighted image
T2WI: T2-weighted image
TBSS: Tract-based spatial statistics
TOF-MRA: Time-of-flight MR angiography
TE: Echo time
TR: Repetition time
TRACULA: TRActs Constrained by UnderLying Anatomy
VBM: Voxel-based morphometry
VOI: Volume of interest
VSRAD: Voxel-based specific regional analysis system for Alzheimer’s disease
WM: White matter
ZTE: Zero-echo time
